# Effectiveness of Platelet-Rich Plasma Injections as Prophylaxis for Recurrent Urinary Tract Infection in Women

**DOI:** 10.3390/jcm12124129

**Published:** 2023-06-19

**Authors:** Yu-Khun Lee, Hann-Chorng Kuo

**Affiliations:** Department of Urology, Hualien Tzu Chi Hospital, Buddhist Tzu Chi Medical Foundation, Buddhist Tzu Chi University, Hualien 970, Taiwan

**Keywords:** platelet-rich plasma, recurrent urinary tract infection, urothelial dysfunction

## Abstract

Purpose: To investigate the therapeutic efficacy of intravesical platelet-rich plasma (PRP) injections as prophylaxis for adult women with recurrent urinary tract infection (rUTI). Methods: This proof-of-concept study enrolled 63 women with rUTI in PRP treatment and control groups after achieving control of the most recent urinary tract infection (UTI) episode. The treatment group included 34 women who received 4 monthly intravesical PRP injections. The control group was made up of 30 women who received continuous antibiotic treatment for 3 months. After the completion of PRP or antibiotic treatment, outpatient follow-up was continued for up to 12 months. Treatment was considered successful if ≤2 UTI episodes occurred during a period of 12 months or ≤1 UTI episode within 6 months; otherwise, the outcome was considered a treatment failure. The frequency of symptomatic UTI episodes before and after PRP treatment was compared with that of the controls. Regression analysis was used to determine the association between potential predictors for a failed treatment outcome. Results: At the study endpoint, 33 PRP and 25 control group patients were available for analysis. After four PRP injections, the frequency of rUTI episodes per month was significantly decreased compared with baseline (0.46 ± 0.27 vs. 0.28 ± 0.30, *p* = 0.047). The PRP treatment success rate was 51.5% (17 of 33) for the PRP group versus 48% (12 of 25) for the control group. The PRP treatment success group had significantly higher voided volume, lower post-void residual volume, and higher voiding efficiency than the PRP treatment failure group. A higher baseline voiding efficacy ≥0.71 was significantly associated with a successful outcome (OR 16.56; *p* = 0.049). Conclusions: The study results revealed that repeat intravesical PRP injections decreased the recurrence rate of UTI within 1 year in women with rUTI. The treatment success rate with intravesical PRP injections for rUTI was about 51.5%, whereas for women with prolonged antibiotic treatment, it was 48.0%. A baseline VE ≥ 0.71 was associated with a better treatment outcome with PRP injections.

## 1. Introduction

Urinary tract infections (UTIs) are a common disease among women, and the majority of them experience recurring episodes within a span of 6 to 12 months [1]. Recurrent UTI (rUTI) is a common but distressing lower urinary tract disease in women. According to an epidemiology survey in 2000, 30% of women experience UTI episodes by the age of 24 years, and more than 50% experience a UTI in their lifetime [2]. Recurrent UTI, including relapses and reinfections, is defined as ≥2 UTI episodes in the past 6 months or ≥3 episodes within the past 1 year [3]. Among women with UTI, 27% experience one recurrence and 3% have two recurrent episodes [4]. Recurrent UTI not only increases public health expenditure but also negatively impacts the quality of life [5]. 

Chronic inflammation of the suburothelium after the resolution of a UTI episode may contribute to persistent urothelial dysfunction and defective barrier function, thus creating conditions that can lead to rUTI [6]. Chronic inflammation disrupts the proliferative function of basal cells, leading to impaired maturation and barrier function of urothelial umbrella cells. This renders urothelial cells more susceptible to bacterial invasion [7]. Hence, the dysfunction of urothelial cells and barrier impairments continue even after the resolution of acute UTI episodes. This can potentially lead to recurrent UTIs following the discontinuation of antibiotic treatment [8]. Restoring normal bladder mucosal barrier relies on enhancing basal cell proliferation in diseased bladders. In this context, eliminating chronic inflammation becomes pivotal as it can promote urothelial regeneration, differentiation, and strengthen the defense mechanism of the affected bladder.

Platelet-rich plasma (PRP) is a platelet-concentrated biological product centrifuged from autologous blood [9]. PRP is rich in several cytokines, chemokines, and growth factors, which enhance wound healing by regulating angiogenesis to increase blood flow and oxygenation in wounds [10,11]. In addition, this plasma is rich in mesenchymal stem cells that play a role in the process of wound healing [12]. These cytokines have the ability to initiate a fresh inflammatory process and aid in resolving unresolved inflammation, effectively alleviating the neurogenic pain caused by the preceding inflammation [13]. Several studies have demonstrated that repeat intravesical PRP injections safely and effectively relieved clinical symptoms in IC/BPS patients [14,15]. A clinical trial of rUTI also showed improvement in urothelial cell proliferation and differentiation after repeat intravesical PRP injections [16]. Based on these data, intravesical PRP injections may recover urothelial health to prevent rUTI. However, more robust evidence is needed to prove the clinical therapeutic efficacy of PRP against rUTI in comparison with conventional antibiotic treatment.

This clinical study used repeat intravesical PRP injections to treat patients with intractable recurrent bacterial cystitis. The aim of this study was to investigate the therapeutic efficacy of repeat PRP injections as prophylaxis in women with rUTI and to search for predictive factors for a successful outcome of repeat PRP injections in this population.

## 2. Materials and Methods

This prospective clinical trial enrolled women with rUTI refractory to conventional antimicrobial treatment to receive intravesical PRP injections or long-term antibiotic treatment. Recurrent UTI was diagnosed based on characteristic clinical symptoms of bacterial cystitis, including micturition pain, frequency, and urgency with or without gross hematuria; pyuria (>10 white blood cell count/high power field), and a positive urine culture at least 2 times within the past 6 months or 3 times within the past 12 months. If the rUTI relapsed within <1 month or the UTI never resolved after antibiotic therapy, the patient was considered to have persistent UTI. A videourodynamic study (VUDS) was routinely performed before treatment to rule out any voiding dysfunction and bladder outlet obstruction. The VUDS was conducted following standard procedures, utilizing a 6-Fr dual-channel catheter and an 8-Fr rectal balloon catheter. A cystometric study was carried out using normal saline at a filling rate of 20 mL/min. All descriptions and terminology in this report adhere to the guidelines set forth by the International Continence Society. The VUDS parameters were recorded, including the first sensation of bladder filling, full sensation, urge sensation, maximum flow rate, voided volume, post-void residual volume (PVR), voiding efficacy (VE), intravesical pressure, detrusor pressure, cystometric bladder capacity, and functional bladder capacity. Patients with voiding dysfunction, bladder outlet obstruction, vesicoureteral reflux, urolithiasis, or diverticulum were excluded from this study.

This study was performed at a medical teaching center in eastern Taiwan. The study was approved by the Institutional Review Board (IRB) and the ethics committee of the hospital (IRB number: 106-121-A). The final analysis also included 6 patients who had received PRP treatment in the pilot study before the initiation of this clinical trial. All patients were informed about the study rationale and procedures, and written, informed consent was obtained before treatment. In the calculation of the power for this study, we estimated the effect size as 0.8 for the experimental groups and control group, and the desired significance level was 0.05. A sample size of 34 for each group is necessary to achieve a power of 0.90. During the study period, we recruited 33 and 30 women for the PRP and antibiotic groups, respectively.

**Figure 1:**
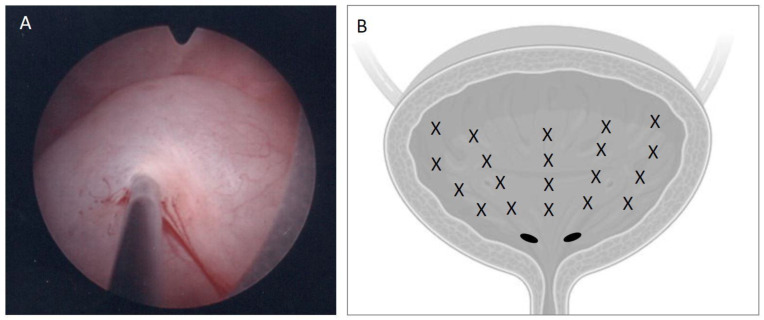
The platelet-rich plasma was injected (**A**) into the suburothelium of the urinary bladder, and (**B**) at 20 sites of the lateral and posterior walls of the bladder.

All PRP treatment group patients underwent a regimen of 4 monthly intravesical PRP injections and were followed up for up to 1 year. The procedure was the extraction of 5 mL of PRP from 50 mL of the patient’s own whole blood and injection at 20 sites equally distributed in the bladder suburothelium (Figure 1), performed under intravenous general anesthesia in the operation room. The PRP was prepared using the procedures detailed in our previous study [14]. The injection was administered using a 23-gauge needle and a 22-Fr rigid cystoscopic injection instrument (Richard Wolf, Knittlingen, Germany). The injection needle was inserted approximately 1 mm into the suburothelium along the posterior and lateral walls. Following the PRP injections, a 14-Fr urethral Foley catheter was kept in place overnight, and patients were discharged the following day. Because of the multiple intravesical injections, all patients received antibiotic treatment for 7 days after each PRP injection to prevent UTI. The first PRP injection was performed after the active UTI episode had been brought under antibiotic control. If any symptomatic UTI occurred during the treatment course, the patients were treated with appropriate antibiotics based on urine culture results until complete recovery.

During the clinical trial period of PRP injection for rUTI, we also enrolled 30 women with rUTI who did not agree to participate in the PRP clinical trial, instead undergoing continuous antibiotic treatment for 3 months after the latest UTI episode, and who served as the control arm. The antibiotics were chosen according to the results of urine culture.

After completing 4 intravesical PRP injections or 3-month antibiotic treatment, patients were scheduled for follow-up at the outpatient clinic every month (for cases of UTI recurrence or persistence) or every 3 months (if no UTI recurrence) for up to 12 months. The primary endpoint of this study was set at 6 months after completion of the 3-month treatment course by PRP injections or antibiotic treatment, and the secondary endpoint was set at 12 months to investigate the durability of PRP treatment. No further antibiotic treatment was given after the first 3 months, and patients were continuously followed up for up to 1 year. Urinalysis was routinely performed at each visit and a urine culture was performed if there was evidence of pyuria in the urinalysis. Antibiotics were prescribed to treat UTI according to the results of urine culture. If ≤2 UTI episodes recurred in the preceding 1 year or ≤1 UTI episode within 6 months, PRP treatment was considered successful; otherwise, the treatment was considered a failure.

The frequency of symptomatic UTI episodes before and after PRP injection treatment or antibiotic treatment was compared using a paired *t*-test. Changes in the number of rUTI episodes after PRP injection or antibiotic treatment for 3 months were compared between the PRP and control groups. We also analyzed the clinical characteristics and VUDS findings at baseline for comparison of the successful and failed PRP treatment outcome groups using a non-parametric Mann–Whitney U test and chi-square test. Binary logistic regression analysis was used to determine the association between potential predictors for a failed treatment outcome. Univariate and multivariate models were used to measure crude and adjusted odds ratios (ORs), respectively, and the 95% confidence interval (CI) for different factors predicting outcomes. All calculations were performed using SPSS (SPSS, Chicago, IL, USA) for Windows, version 10.0. Statistical significance was considered to be a value of *p* < 0.05.

## 3. Results

This study enrolled 34 women with rUTI who received intravesical PRP injections and 30 women with rUTI who received continuous antibiotic treatment for 3 months. The baseline demographics of the PRP and control groups are listed in Table 1. The mean age, rate of rUTI and persistent UTI, and medical comorbidities were similar between the PRP and control groups. Patients who received PRP treatment had more UTI episodes per year.

At the study endpoints, 33 patients in the PRP group and 25 in the control group were available for follow-up for at least 6 months. In the PRP group, 29 patients could be followed up at 12 months and the other 4 patients could only be followed up for 6 months. In the control group, 17 patients were followed up at 12 months and 8 at 6 months. After 4 PRP injections, the frequency of rUTI episodes per month was significantly decreased compared with baseline (0.46 ± 0.27 vs. 0.28 ± 0.30, *p* = 0.047). Compared with the control, more patients in the PRP group had a successful result at the 12-month follow-up (*p* = 0.037). However, no significant difference in the decrease in rUTI episodes was noted for the PRP group compared with the control group (0.46 ± 0.28 compared to 0.21 ± 0.27) (*p* = 0.352). The treatment success rate was 51.5% (17 of 33) for PRP patients versus 48% (12 of 25) for the control group during the follow-up period. No febrile acute UTI or urine retention was reported after PRP treatment. At the 6- and 12-month follow-ups, the treatment success rate remained similar within the PRP and control groups (Table 2).

Of the 33 PRP patients, 17 had a successful treatment outcome, and 16 experienced treatment failure. Table 3 shows the baseline VUDS parameters between the treatment success and failure groups. After PRP treatment, the treatment success group had a significantly higher decrease (from 0.39 ± 0.26 to 0.12 ± 0.12) in the number of UTI episodes per month compared with the failed treatment group (from 0.51 ± 0.27 to 0.53 ± 0.32) (*p* = 0.005). A comparison of baseline VUDS parameters showed that the treatment success group had significantly higher voided volume, lower PVR, and higher VE than the failed treatment group. Binary logistic regression analysis on the multivariate data revealed that a higher baseline VE (≥ 0.71) was significantly associated with a successful outcome (OR 16.56; 95% CI, 1.02–269.4; and *p* = 0.049).

**Figure 2:**
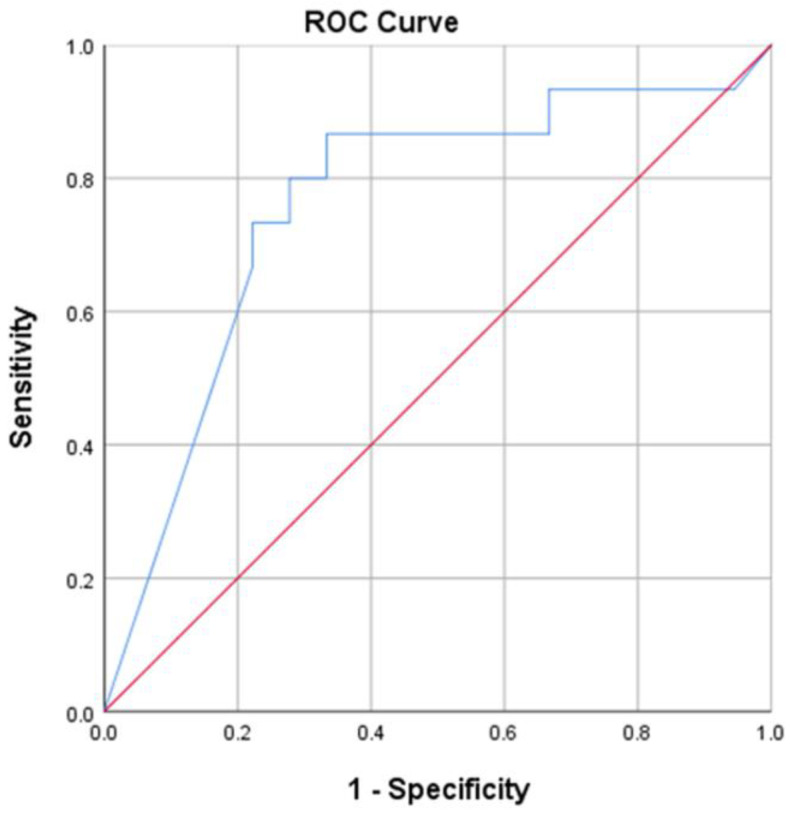
Receiver operating characteristic curve for pretreatment voiding efficacy based on the successful outcome after platelet-rich plasma (PRP) treatment; area under the curve = 0.761 and *p* = 0.011.

With the successful outcome after PRP treatment at the study endpoint, a cutoff value of 0.71 for pretreatment VE was obtained through receiver operating characteristic curve analysis. There were 19 (55.9%) patients with pretreatment VE ≥ 0.71 and 15 (44.1%) patients below this point. The cutoff value sensitivity and specificity were 86.7% and 66.7%, respectively. As shown in Figure 2, the area under the curve was 0.761 (95% CI 0.587–0.935; *p* = 0.011).

No significant difference in baseline comorbidities was found between the treatment success and failure groups. In addition, no significant differences between the treatment success or failure groups were observed for the following: mean age (65.3 ± 8.92 years vs. 73.0 ± 5.92 years; *p* = 0.091); persistent UTI (5 patients, 29.4% vs. 10 patients, 62.5%; *p* = 0.371); diabetes mellitus (DM, 3 patients, 17.6% vs. 9 patients, 56.3%; *p* = 0.152); neurogenic voiding dysfunction (NVD, none vs. 6 patients, 37.5%; *p* = 0.999); and hysterectomy (6 patients, 35.3% vs. 3 patients, 18.8%; *p* = 0.434), respectively. However, a comparison of the UTI episode rate after PRP treatment showed that patients without NVD and DM had a significantly higher success rate than patients with NVD and DM (Table 4). Adverse events after PRP injections included mild hematuria after injections in 4 women, which subsided spontaneously without further treatment. No acute febrile pyelonephritis or bladder pain complaints were reported.

## 4. Discussion

Recurrent urinary tract infection (rUTI) is among the most prevalent conditions associated with female lower urinary tract dysfunction [2]. Our previous studies showed that urothelial barrier impairment and urothelial cell apoptosis were remarkable in the bladders of women with rUTI, and the chronic inflammation of the bladder urothelium might be the underlying pathophysiology of rUTI [6]. The persistence of high levels of urinary nerve growth factor is detectable in patients with rUTI, suggesting that chronic inflammation in the bladder wall may exist for a period of time after the subsidence of the initial UTI episode [17,18]. Patients with recurrent urinary tract infection (rUTI) were found to exhibit deficiencies in the expression of proteins related to urothelial cell proliferation, cytoskeleton, and barrier function [19].

A normal bladder urothelium has an intact barrier against urinary solutes and bacteria. The regulation of urothelial integrity relies on the proliferation and differentiation of stem cells and progenitor cells within the urothelial basal layers. Sonic hedgehog (SHH) protein is a marker for urothelial basal cells, and increased expression of both SHH and CD34 is noted with urothelial injury in the bladder [20]. Within 24 h of uropathogenic *Escherichia coli* (UPEC) infection, basal cell proliferation causes thickening of the urothelial cell layers. As a result of this process, there is an increased exfoliation and desquamation of the infected apical cells, leading to the elimination of intracellular bacteria and subsequent resolution of the urinary tract infection [21].

In a healthy bladder, the urothelium regenerates rapidly within hours of a bacterial invasion [22]. This reaction promotes the exfoliation of the apical urothelial cells and expels the bacteria in these cells [12]. However, this defense mechanism may be insufficient in cases of UPEC in patients with impaired urothelial regeneration, resulting in intracellular bacterial colonization emerging from the unshed cells [23]. The persistence of UPEC in bladder tissue can lead to recurrent episodes of acute cystitis through a complex interplay of dynamic host-pathogen interactions [24]. Restoring the function of urothelial regeneration and differentiation is crucial for effectively eliminating bacteria that may be harbored within defective urothelial cells.

In an ultrastructural bladder urothelium study in women with rUTI, deficits in the expression of proteins of urothelial cell proliferation, maturation, and barrier function were noted [19]. In addition, high urinary nerve growth factor has been detected in the urine of women with rUTI, even after symptomatic resolution of the acute UTI episode, suggesting that chronic inflammation persists in the bladders of these patients [25]. Therefore, urothelial dysfunction and barrier deficits persist after acute UTI episodes, as the bladders still had immature urothelium, various ultrastructural deficits, and elevated urinary inflammation, which may result in rUTI after antibiotic treatment is discontinued [8]. Intravesical PRP injections have been shown to improve basal cell proliferation in diseased bladders and increase uroplakin expression, which are crucial to restoring the normal bladder mucosal barrier [26]. In this regard, eliminating chronic inflammation by repeated PRP injections may improve urothelial regeneration and differentiation and rebuild the defense mechanism of the diseased bladder.

In both orthopedics and sports medicine, PRP has been widely used to enhance postoperative wound recovery [11]. Several types of growth factors may be found in PRP, including platelet-derived growth factor, epidermal growth factor, and transforming growth factor. Hence, PRP therapy is based on the proliferation of cells and their migration to the damaged site, subsequent cell differentiation, and angiogenesis, leading to early wound healing [10,12].

Repeat intravesical PRP injections have proven benefits in IC/BPS patients, improving their clinical symptoms [14]. The clinical impact of intravesical PRP injections on IC/PBS is additionally evident through alterations in urinary levels of vascular endothelial growth factor and cytokines [15]. These findings offer evidence of altered urinary protein expression, supporting the therapeutic efficacy of PRP in treating IC/BPS. This suggests that intravesical PRP treatment may enhance urothelial health by stimulating cell proliferation, migration, differentiation, and angiogenesis in patients who are unresponsive to conventional therapy for IC/BPS [9,27]. 

Recently, the authors conducted a pilot study using autologous PRP in the treatment of recurrent bacterial cystitis. The findings demonstrated that repeated intravesical PRP injections could enhance urothelial cell proliferation by increasing CD34 expression. Moreover, these injections improved the differentiation of urothelial basal cells into mature apical cells, as indicated by the characteristic expression of CK20 and the functional proteins M2 and M3 [16]. The present study also showed that repeat intravesical PRP injections may reduce the UTI recurrence rate in women with rUTI. The results of both studies provide robust and objective evidence of the therapeutic effects of PRP in women with rUTI.

The present study showed that a higher VE (0.71) is associated with a successful outcome of PRP treatment. Patients with voiding dysfunction and bladder outlet obstruction were excluded from this study. However, the patients with unsuccessful treatment outcomes at the study endpoint had significantly higher PVR (180.4 ± 243.4 mL) and a lower VE (0.51 ± 0.34) than those in the successful group, suggesting that patients with treatment failure might have subclinical voiding dysfunction. A high PVR was significantly associated with rUTI in male patients, even in patients without lower urinary tract symptoms [28]. A PVR volume of 180 mL or higher exhibited the highest specificity and sensitivity in predicting bacteriuria in asymptomatic adult men [29]. The significance of high PVR in rUTI among women remains a subject of debate. However, postmenopausal women experiencing rUTI demonstrated a significantly elevated PVR and diminished urine flow rate compared to age-matched controls [30,31]. However, in young, healthy, nonpregnant women, no difference in PVR was noted between rUTI patients and controls [32]. According to most current guidelines, an elevated PVR is considered an independent risk factor for recurrent urinary tract infections (rUTI) in women; therefore, PVR should be measured before the management of UTI in women [33]. 

Our analysis of patient characteristics showed that patients aged >75 years and those with persistent UTI, DM, or NVD had a lower success rate for PRP treatment. Although only DM and NVD were statistically significant, patients with these risk factors should be treated with prolonged pharmacologic therapy with antibiotics. Previous studies have indicated that normal bladders sustain suburothelial circulation throughout bladder filling, even with significant stretching of the bladder wall. Temporary decreases in blood supply are only observed during voiding [34]. However, in bladders with chronic inadequate drainage or urinary retention, there may be a prolonged reduction in blood flow during the filling phase, resulting in repeated episodes of ischemia and reperfusion [35]. The reduction in bladder wall oxygen tension also altered urothelial barrier function by decreasing urothelial cell proliferation and differentiation [36]. Although PRP could increase urothelial proliferation and differentiation, the therapeutic effect may be influenced by persistent bladder ischemia and oxidative stress.

Although intravesical PRP injections may benefit the recovery of urothelial function in patients with rUTI, the therapeutic effect is still unsatisfactory. A previous clinical trial enrolled 22 patients with rUTI and showed a treatment success rate of 63.6%. By contrast, the present study assessed a larger population of 34 women with rUTI but found a lower success rate (51.5%). Compared with the women who received continuous antibiotic treatment for 3 months in this study, however, PRP treatment was not inferior to treatment with antibiotics. In daily clinical practice of rUTI treatment, not all women are able to undergo antibiotic treatment for 3 months; therefore, PRP could be an alternative prophylactic treatment against rUTI in women. This study is a proof-of-concept trial, which lacks a randomized control arm based on injecting a placebo. Nevertheless, this pilot study provided valuable experience of a novel treatment for rUTI in women. Further control studies are necessary to prove the actual therapeutic efficacy of PRP treatment on rUTI.

The limitations of this study are the small number of patients, non-randomization of patient groups, and the complexity of both PRP and control groups receiving antibiotic treatment, with antibiotic prophylaxis for 7 days after each PRP injection to prevent UTI. Women with persistent UTI were also included in this study, which may have decreased the PRP treatment success rate because these patients may have residual bacterial colonies in the bladder wall, which may not be eradicated without prolonged antibiotic therapy. In addition, fewer patients with persistent UTI received prolonged antibiotic treatment, which may also result in a bias in the treatment outcome for the control group.

The success rate of PRP and long-term antibiotic treatment are comparable in this study. This result indicates that repeat PRP injection treatment can also achieve a satisfactory treatment outcome, equivalent to that of antibiotic treatment for 3 months. However, this proof-of-concept clinical trial has demonstrated that PRP injection could improve the bladder urothelial barrier function and eliminate recurrent bacterial inoculation and infection. This study provides evidence for future research on the prevention of recurrent UTI. Although active treatment was provided for these women with rUTI, only half of them could have a successful outcome, suggesting that long-term antibiotic treatment might be necessary for patients with recurrent UTI. The cost of four PRP preparations and 3-month antibiotic treatment should be balanced with the treatment outcome. 

## 5. Conclusions

The results of this proof-of-concept study revealed that repeat intravesical PRP injections may decrease the recurrence rate of UTI in women with rUTI within 1 year of follow-up. However, the treatment success rate was similar between the PRP injection and 3-month antibiotic treatment groups. A higher VE at baseline was associated with a successful therapeutic outcome of PRP treatment.

## Figures and Tables

**Table 1:** Baseline demographics of the platelet-rich plasma (PRP) treatment and medical treatment (control) groups.

Baseline	PRP Injection (N = 34)	Control (N = 30)	*p*-Value *
Age (year)	68.59 ± 8.72	63.17 ± 15.26	0.182
History of recurrent UTIs	6.39 ± 7.41	4.17 ± 4.71	0.041
Recurrent UTI (≤3/year)	19 (55.9%)	22 (75.9%)	0.097
Persistent UTI (>3/year)	15 (44.1%)	7 (24.1%)	
Diabetes mellitus	13 (38.2%)	10 (33.3%)	0.683
Hypertension	23 (67.6%)	3 (10.0%)	0.000
CVA	2 (5.9%)	1 (3.3%)	1.000
Cervical cancer s/p radical hysterectomy	1 (2.9%)	2 (6.7%)	0.596
Post hysterectomy	10 (29.4%)	4 (13.3%)	0.120
History of urolithiasis	3 (8.8%)	0 (0.0%)	0.241
NVD	6 (17.6%)	4 (13.3%)	0.635

Abbreviations: UTI, urinary tract infection; CVA, cerebrovascular accident; NVD, neurogenic voiding dysfunction. * non-parametric Mann–Whitney U test and chi-square test.

**Table 2:** Treatment outcome and decreased episodes of urinary tract infection (UTI) between the platelet-rich plasma (PRP) and control groups.

	PRP Injection (N = 33)	Control (N = 25)	*p*-Value
Success rate (UTI ≤ 1/6 M) *	4/4 (100%)	7/8 (87.5%)	1.000
Success rate (UTI ≤ 2/12 M)	13/29 (44.8%)	5/17 (29.4%)	0.037
Pre-PRP/antibiotics UTI (episode/M)	0.46 ± 0.27	0.46 ± 0.28	0.980
Post-PRP/antibiotics UTI (episode/M)	0.28 ± 0.30 **	0.21 ± 0.27	0.229
UTI reduction (episode/M)	−0.18 ± 0.29	−0.24 ± 0.32	0.413

* Patients were only available for follow-up at 6 months; ** *p* = 0.047.

**Table 3:** Baseline characteristics and comorbidities between platelet-rich plasma (PRP) patients with successful and failed treatment outcomes.

	Success (N = 17)	Failure (N = 16)	*p*-Value *
FSF (mL)	133.8 ± 32.1	149.5 ± 96.3	0.787
FS (mL)	210.1 ± 54.2	215.9 ± 125.3	0.820
US (mL)	257.7 ± 78.0	220.7 ± 110.1	0.247
Qmax (mL/s)	15.1 ± 6.7	10.5 ± 8.01	0.078
Volume (mL)	269.2 ± 133.4	165.1 ± 124.3	0.043
PVR (mL)	36.2 ± 67.5	180.4 ± 243.4	0.007
VE	0.86 ± 0.28	0.51 ± 0.34	0.002
Pves (cmH_2_O)	30.5 ± 24.1	39.0 ± 20.1	0.105
Pdet (cmH_2_O)	17.9 ± 9.20	24.0 ± 21.0	0.678
CBC (mL)	305.4 ± 120.4	357.5 ± 253.7	0.925
FBC (mL)	368.5 ± 140.7	288.9 ± 198.6	0.224

Abbreviations: FSF, first sensation of bladder filling; FS, full sensation; US, urge sensation; Qmax, maximum flow rate; Volume, voided volume; PVR, post-void residual volume; VE, voiding efficacy; Pves, intravesical pressure; Pdet, detrusor pressure; CBC, cystometric bladder capacity; FBC, functional bladder capacity. * non-parametric Mann–Whitney U test.

**Table 4:** Effect of patient characteristics on recurrent urinary tract infection (UTI) episodes and success rate after platelet-rich plasma (PRP) treatment.

**Patient** **Characteristics**	**N**	**Pre-PRP** **UTI/Month**	**Post-PRP** **UTI/Month**	**UTI** **Reduction**	**Success** **Rate**
Age < 75 Age ≥ 75	25 8	0.43 ± 0.27	0.22 ± 0.25	−0.20 ± 0.32	15 (60.0%)
		0.56 ± 0.26	0.45 ± 0.37	−0.10 ± 0.13	2 (25.0%)
		*p* = 0.159	*p* = 0.054	*p* = 0.343	*p* = 0.118
Recurrent UTI Persistent UTI	18 15	0.34 ± 0.25	0.18 ± 0.19	−0.16 ± 0.34	12 (66.7%)
		0.60 ± 0.22	0.40 ± 0.35	−0.20 ± 0.22	5 (33.3%)
		*p* = 0.001	*p* = 0.055	*p* = 0.550	*p* = 0.056
NVD Non-NVD	6 27	0.55 ± 0.33	0.60 ± 0.31	0.05 ± 0.27	0
		0.44 ± 0.26	0.21 ± 0.25	−0.23 ± 0.27	17 (63.0%)
		*p* = 0.510	*p* = 0.006	*p* = 0.030	*p* = 0.007
DM Non-DM	12 21	0.55 ± 0.30	0.42 ± 0.35	−0.13 ± 0.30	3 (25.0%)
		0.40 ± 0.24	0.20 ± 0.24	−0.21 ± 0.29	14 (66.7%)
		*p* = 0.144	*p* = 0.034	*p* = 0.216	*p* = 0.021
ATH Non-ATH	9 24	0.55 ± 0.33	0.23 ± 0.28	−0.32 ± 0.40	6 (66.7%)
		0.42 ± 0.23	0.29 ± 0.31	−0.13 ± 0.23	11 (45.8%)
		*p* = 0.372	*p* = 0.527	*p* = 0.232	*p* = 0.438

Abbreviations: UTI, urinary tract infection; NVD, neurogenic voiding dysfunction; DM, diabetes mellitus; ATH, abdominal hysterectomy.

## Data Availability

Not applicable.
